# Fick's second law transformed: one path to cloaking in mass diffusion

**DOI:** 10.1098/rsif.2013.0106

**Published:** 2013-06-06

**Authors:** S. Guenneau, T. M. Puvirajesinghe

**Affiliations:** 1 Institut Fresnel, UMR CNRS 7249, Aix Marseille Université, Campus de St Jérôme, Marseille Cedex 20 13397, France; 2 Institut Paoli-Calmettes, UMR INSERM 1068, UMR CNRS 7258, Aix Marseille Université, Marseille, France

**Keywords:** cloaking, effective medium, finite elements, mass diffusion

## Abstract

Here, we adapt the concept of transformational thermodynamics, whereby the flux of temperature is controlled via anisotropic heterogeneous diffusivity, for the diffusion and transport of mass concentration. The *n*-dimensional, time-dependent, anisotropic heterogeneous Fick's equation is considered, which is a parabolic partial differential equation also applicable to heat diffusion, when convection occurs, for example, in fluids. This theory is illustrated with finite-element computations for a liposome particle surrounded by a cylindrical multi-layered cloak in a water-based environment, and for a spherical multi-layered cloak consisting of layers of fluid with an isotropic homogeneous diffusivity, deduced from an effective medium approach. Initial potential applications could be sought in bioengineering.

## 1. Introduction

This report aims to target already-existing systems that could enable the use of cloaking concepts in order to achieve control of three-dimensional processes, using coated spheres consisting of concentric layers of homogeneous isotropic diffusivity. Various applications already implicate the use of concentric bilayered vesicles, one example being liposomes used for drug delivery [1]. Liposomes are concentric bilayered vesicles in which an aqueous volume containing a water-soluble drug is enclosed by a membranous lipid bilayer composed of natural or synthetic phospholipids. One popular type of liposomes, known as the stealth liposomes [2], are highly stable, long-circulating liposomes whereby polyethylene glycol has been used as the polymeric steric stabilizer [3]. Stealth and other liposomes use the concept of ‘invisibility’ in order to hide and evade the immunosystem by coupling water-soluble polymers to the lipid heads. Therefore, the polymer part of the molecule is dissolved in the aqueous environment, thus masking the liposomes from immune cells in the blood [4]. Other alternative applications to liposomes are nanoparticles based on solid lipids (SLNs). These are composed of SLNs stabilized with an emulsifying layer in an aqueous dispersion. This has benefits such as drug mobility. The release of the drug-enriched core of SLN is based on Fick's first law of diffusion [5–7].

Another similar idea to the one presented in this paper is the concept of optical transparency, resulting from the use of preparative reagents for the subcellular localization of fluorescently labelled tissues and organisms [8]. High-resolution imaging techniques, such as laser scanning microscopy, are able to provide high-resolution images of biological samples. However, the resolution of imaging whole organisms, such as embryos by fluorescently labelling certain components, becomes somewhat distorted owing to the biological samples containing optically opaque regions. These opaque components are able to transmit, reflect, scatter as well as absorb light, which can lead to image distortions. Therefore, certain commercially or non-commercially available reagents are available that are known as optical-clearing reagents [8,9] and can render tissues and organisms transparent.

Here, we suggest a novel application to the fast-growing research area of cloaking, whereby a better control of light can be achieved through transformational optics, following the pioneering theoretical works of Pendry *et al.* [10] and Leonhardt [11], to diffusion processes in biophysics. Pendry *et al.* [10] demonstrate the possibility of designing a cloak that renders any object inside it invisible to electromagnetic radiation (using the covariant structure of Maxwell's equations), whereas Leonhardt [11] concentrates on the ray optics limit (using conformal mappings in the complex plane for Schrödinger's equation). In both cases, the cloak consists of a meta-material whose physical properties (permittivity and permeability) are spatially varying and matrix-valued. This route to invisibility is reminiscent of the work of Greenleaf *et al.* [12] in the context of electrical impedance tomography. Interestingly, the isomorphism between the anisotropic conductivity and thermostatic equations makes it possible to control the pathway of heat flux in a stationary setting, as observed by Fan *et al.* [13] (see [14] for analogous cloaking in electrostatics) and experimentally validated by Narayana & Sato [15]. However, time plays an essential role in diffusion processes, and manipulation of heat flux through anisotropic diffusivity requires greater care in a transient regime [16,17]. Interestingly, anisotropic diffusion is a well-known technique in computer vision [18,19] aiming to reduce image noise without removing significant parts of the image content, typically edges, lines or other details that may be important in the interpretation of the image [20]. Spatio-temporal differential equations of a reaction–diffusion type also appear in organogenesis models for the developments of limbs, lungs, kidneys and bones [21].

The mathematical model described in this report is based on Fick's laws of diffusion, derived by Fick [22], which describes diffusion processes governing various contexts (conduction of electricity, heat, concentration of chemical species, etc., and even grey-scale image in computer vision). Here, the diffusion coefficient is spatially varying (heterogeneous) and matrix-valued (anisotropic). We show numerically that control can be achieved in three-dimensional processes (with a focus here on bioengineering applications), using coated spheres consisting of concentric layers of homogeneous isotropic diffusivity, which mimic certain anisotropic heterogeneous diffusivity. Previous studies have only shown the control of diffusion processes with two-dimensional transformational thermodynamics [13,15,16]. Using a similar strategy, we extend previous works to three-dimensional diffusion processes and also discuss the issue of convection.

## 2. Results and discussion

**Figure 1.**
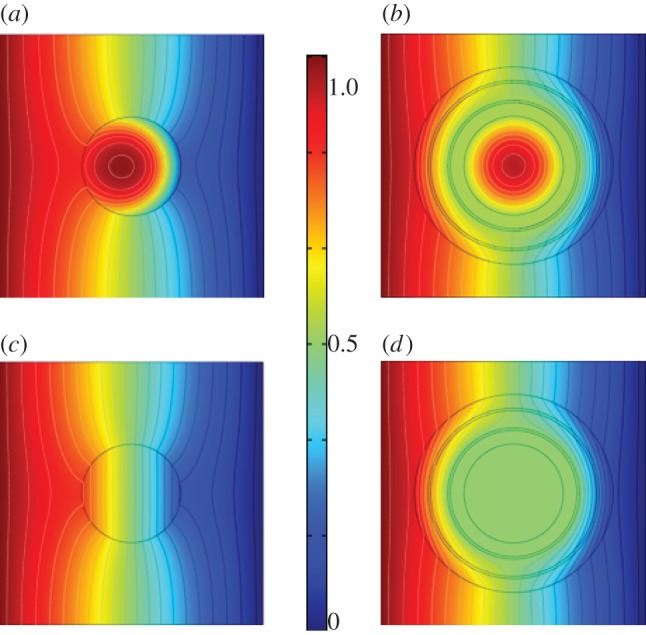
Two-dimensional simulation for diffusion of chemical species' concentration: concentration is normalized to 1 mol m^−3^ on the left boundary with a flux boundary condition on right boundary with mass transfer coefficient of 5 m s^−1^, and symmetry boundary conditions on top and bottom; two time point (*t* = 1 × 10^–6^ s (*a*,*c*); *t* = 1.5 × 10^–5^ s (*b*,*d*)) simulations of mass diffusion in surrounding medium with diffusion constant of 2.1 × 10*^−^*^9^ m s^−2^ (CO_2_–water) of a circular nano-size particle (nanobody) with a diameter of 1.5 × 10*^−^*^8^ m of diffusion constant 1.9 × 10^−11^ m s^−2^ (POPC-dehydrated). (*b,d*) Application of a cloak, surrounding the nanobody, which is of inner radius 1.5 × 10^−8^ m and outer radius 3.0 × 10^−8^ m and consists of five concentric layers. The first, third and fifth layers from the inside the cloak outwards have a diffusivity of 4.586 × 10*^−^*^10^ m s^−2^ (sucrose in water) and respective thicknesses 4.25 × 10^−9^, 5.25 × 10^−9^ and 4 × 10^−9^ m. The second and fourth layers have diffusivities of 11 × 10*^−^*^6^ m s^−2^ (gas ethanol–air) and 8.4 × 10^−9^ m s^−2^ (liquid CO_2_–methanol) and identical thickness 7.5 × 10*^−^*^10^ m. Note that in (*b*,*d*) isovalues of concentration (black curves) are bent around the nanobody, whereas they remain aligned outside the cloak. (Online version in colour.)

Recent work used a change of coordinates in the time-dependent heat equation [23] to achieve a marked enhancement in the control of heat fluxes in two-dimensional media described by an anisotropic heterogeneous conductivity [16]. However, it has been known since the work of Fick [22] that there is a deep analogy between diffusion and conduction of heat or electricity: because of Fick's work, diffusion can be described according to the same mathematical formalism as Fourier's law for heat conduction, or Ohm's law for electricity. We would like to use similar analogies between diffusion of heat and concentration of chemical species to propose an original strategy towards cloaking in bioengineering/chemical engineering. A possible application is shown in figure 1, through the creation of different layers. This concept has already been used to a certain extent in multi-vesicular liposomes that consist of bilayers of phospholipids. Using the diffusivity values published for a certain lipid-conjugated drug (1.9 × 10*^−^*^11^ m s^−2^ for dehydrated 1-palmitoyl-2-oleoyl-sn-glycero-3 phosphocholine (POPC)) and coating these nano-sized particles (30 nm) with different layers of sucrose (diffusivity of 4.586 × 10*^−^*^10^ m s^−2^) and also thin layers containing substances of higher diffusivities, results in the initial particle becoming invisible in the chemical environment (diffusivity value of 2.1 × 10^−9^ × m s^−2^), which is used to represent an environment such as blood whose main constituent plasma is essentially composed of water [24,25]. This can be seen with the comparison of the distribution of concentration without the cloak (figure 1*a,c*) and with the cloak (figure 1*b*,*d*). The consequences of the additional layers are more prominent at longer time points compared with shorter time points, *t* = 1.5 × 10^–5^ s (figure 1*c*,*d*) compared with *t* = 1 × 10^–6^ s (figure 1*a*,*b*). Here, the effect of the different layers aids in maintaining high and uniform concentrations of a substance in the centre of the liposome for longer periods of time. This could have advantages in increasing drug stability for longer circulation times.

Potential fabrication of this alternative liposome would involve additional procedures to the classical liposome fabrication steps currently used. More specifically, chloroform or chloroform–methanol or mixing with the lipid and hydrophobic organic solvents could still be used for the formation of vesicles. The appropriate removal of solvents involving rotary evaporation for extended time periods could still be used. This could be followed by frozen storage before consequent steps. Second, replacing the classical hydration step by using an aqueous medium but with a higher concentration of sucrose compared with traditional sucrose concentrations (with mixtures of glycine or alanine, which share the same diffusion coefficients and are also traditionally used for coating drugs), would allow the creation of sucrose layers during the formation of micelles. Choosing appropriately sized vesicles would involve sonication techniques and analysis using techniques used for vesicular structures such as dynamic light scattering equipment and transmission electron microscopy.

Layers containing the gaseous phase or an appropriate replacement of a high diffusivity value (in the range of 10^−6^ to 10^−5^ m s^−2^) would be the most complicated steps in the fabrication process. In practice, it would be more feasible to replace the gaseous layers used in the numerical stimulations by media of similar diffusivity values, owing to difficulties in initial fabrication and stability or maintenance of these layers.

It should be noted that the examples of specific substances chosen for the simulations can easily be replaced by alternative appropriate substances with the same diffusion coefficient values. In addition, if potential cloaking applications are non-objectionable to the use of chloroform, then it should be noted that this replacement has already been calculated to be a good substitute for the sucrose layers (layer one, three and five). This is shown in the electronic supplementary material, figure S1, wherein there is improved cloaking as the isovalue curves for concentration outside the multi-layered structure are nearly perfectly aligned (figure 1*b*,*d*) in contradistinction to what can be observed in figure 1*a*,*c* for a microparticle not surrounded by a cloak. Electronic supplementary material, figure S2 shows that the maximum concentration within the microparticle is always lower when it is surrounded by the cloak (and that its variation is markedly reduced because of the cloak). This is demonstrated by calculating the concentration at all points along a line passing through the centre of the nanoparticle without (see the electronic supplementary material, figure S2*a*,*c*) and with (see the electronic supplementary material, figure S2*b*,*d*) a surrounding cloak. Note that this is achieved with simply five concentric layers, three of which have same diffusivity. A similar type of profile for concentration can be observed in the electronic supplementary material, figure S4, for a three-dimensional cloak with 20 layers described in the electronic supplementary material, figure 3, whose design requires a detailed analysis of the *n*-dimensional-transformed Fick's equation. This is the object of §2.1.

### 2.1. *n*-Dimensional-transformed convection–diffusion equation

We consider the convection–diffusion equation that is a parabolic partial differential equation combining the diffusion equation and the advection equation. This equation describes physical phenomena where particles or energy (or other physical quantities) are transferred inside a physical system owing to two processes: diffusion, which results in mixing and transport of chemical species without requiring bulk motion (it is a random walk of particles/molecules towards certain equilibrium state, i.e. homogeneous distribution of chemical species inside a region); and convection, whereby collective movements of ensembles of molecules take place (usually in fluid) which, in essence, use bulk motion to move particles from one place to another place [26]. In its simplest form (when the diffusion coefficient and the convection velocity are constant and there are no sources or sinks), the convection–diffusion equation in a domain *Ω* (with a chemical source outside) can be expressed as [27]2.1
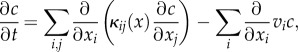
where *c* represents the mass concentration (in biochemistry) evolving with time *t* > 0, *κ* is the chemical diffusion in units of m^3^ s^–1^ and *v* is the velocity field. We note that Fick's equation is written in a general form, where *x* = (*x*_1_, … ,*x_n_*) is a variable in an *n*-dimensional space. Accordingly, sums stretch from *i*, *j* = 1, … *n* (here applications are sought in two- and three-dimensional spaces, so *n* = 2 or 3). It is customary to put matrix 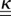 in front of the spatial derivatives when the medium is homogeneous. However, here we consider a heterogeneous (possibly anisotropic) medium; hence, the spatial derivatives of 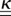 might suffer some discontinuity (mathematically, partial derivatives are taken in distributional sense [28]; hence, transmission conditions ensuring continuity of the heat flux 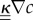 are encompassed in (2.1)). Physically, the diffusion flux 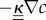 measures the amount of substance that will flow through a small volume during a short time-interval (mol m^−3^ s^–1^).

Upon a change of variable *x* = (*x*_1_, *x*_2_, *x*_3_) → *y* = (*y*_1_, *y*_2_, *y*_3_) described by a Jacobian matrix **J** such that *J_ij_* = *∂y_i_*/*∂x_j_*, (2.1) takes the form:2.2
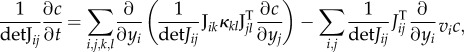
where 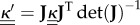 and *v′* = det(**J**)*^−^*^1^**J**^T^*v* are the transformed diffusivity and velocity, respectively.

### 2.2. Jacobian matrix and transformed diffusivity for cloaking

Let us now consider the following transform [12]2.3
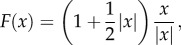
where 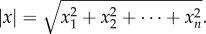 This function is smooth except at point *O* = (0, … ,0). It blows up the point *O* to the hypersphere of radius *|x|* = 1, while mapping the hypersphere of radius *|x|* = 2 to itself. Moreover, *F*(*x*) = *x* at the boundary *|x|* = 2.

Defining the Jacobian matrix **J** as *J_ij_* = ∂*F_i_*/∂*x_j_*, we find2.4
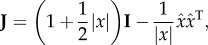
where **I** is the *n* × *n* identity matrix and 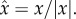 This Jacobian is well defined everywhere except at *x* = 0.

We note that **J** is symmetric, 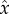 is an eigenvector with eigenvalue 1/2 and 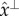 is an *n* − 1 dimensional eigenspace with eigenvalue 1/2 + 1/*|x|* in a space of dimension *n*.

The determinant of the Jacobian follows easily:2.5
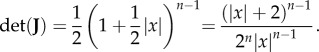


There are two cases of practical importance: *n* = 2, for which writing 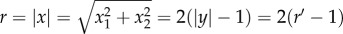 with obvious notations, one can see that the eigenvalues of the matrix of transformed diffusivity 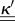 behave like *r* and *r*^−1^ as *r* → 0; *n* = 3 for which writing 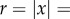 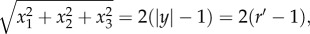 one can see that the matrix of transformed diffusivity 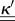 has one eigenvalue that behaves like *r*^2^ and two behave like *r*^0^ as *r* → 0. Interestingly, for *n* ≥ 4, one eigenvalue of 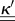 behaves like *r^n^*^−1^ and the remaining *n*−1 behaves like *r^n^*^−3^. This might be of use in computer vision [18]. This shows that only the case *n* = 2 leads to a singular matrix of diffusivity 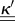 at the inner boundary of the cloak (the circumferential eigenvalue becomes infinite), a fact already noticed in the context of cloaking for electric impedance tomography [12,29]. However, this matrix is always degenerate at the inner boundary of the cloak, irrespective of the space dimension. Similarly, *v′* is a null vector on the inner boundary of the cloak only in space dimension 2. This analysis provides evidence that spherical cloaks should be easier to construct than circular cloaks.

The parameters of the biocloak need to be further analysed in both polar and spherical coordinates in order to simplify the cloak's design and the numerical implementation.

### 2.3. On the choice of reduced parameters for a biocloak in polar and spherical coordinates

We first note that if we multiply both sides of (2.2) by detJ*_ij_* and let detJ*_ij_* inside the partial space derivatives, then we retrieve the usual form of the convection–diffusion equation, albeit with anisotropic coefficients. We realize this is not legitimate in general as by doing so we add an extra term 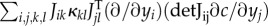 in (2.2), but we numerically checked that this term can be sufficiently small that it does not significantly affect the solution of the transformed equation (2.2). Physically, this manipulation results in preserving the direction of the diffusion flux 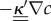 (because detJ*_ij_* is a scalar). However, it affects its continuity (because it is heterogeneous). Such a manipulation is known in the transformational optics community to lead to transformed equations with reduced parameters [30]. We now observe that from the function *F*(*r*) = *R*_1_ + *r*(*R*_2_−*R*_1_)/*R*_2_ counterpart of (2.3), wherein *R*_1_ = 1 and *R*_2_ = 2, in polar (*r*, *θ*) (resp. spherical (*r*, *θ*, *ϕ*)) coordinates, which blows up a point *O* to the disc (resp. the sphere) of radius *R*_1_ and maps the disc (resp. the sphere) of radius *R*_2_ to itself in polar (*r’*, *θ’*) (resp. spherical (*r’*, *θ’*, *ϕ′*)) coordinates, the transformed diffusivity can be expressed for a cylindrical cloak as:2.6
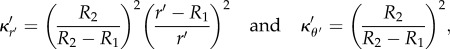
and for a spherical cloak as2.7
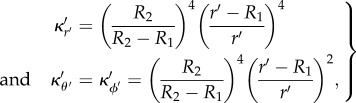
where *R*_1_ and *R*_2_ are the interior and the exterior radii of the cloak so designed. One can note that when *r*′ tends to *R*_1_, 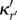 goes to zero and 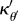 remains constant in (2.6), whereas 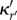, 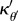 and 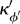 all go to zero in (2.7), albeit with different speeds. This means that thanks to the reduced coefficients the matrix of transformed diffusivity 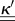 now has one eigenvalue that behaves like *r*^2^ and one like *r*^0^ as *r* → 0 in the cylindrical case, i.e. we no longer have an eigenvalue that blows up on the inner boundary of the cloak. Likewise, we now have one eigenvalue that behaves like *r*^4^ and two like *r*^2^, instead of one behaves like *r*^2^ and two like *r*^0^ when *r* tends to zero, if we use reduced parameters in the spherical case. Thus, reduced parameters are an obvious choice in the cylindrical case, because 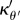 is a constant in (2.6), and were implemented for thermal cloaks in [16,17]. However, in spherical case, 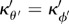 are no longer constant in (2.7). Nevertheless, we need to use such reduced coefficients to get rid of the coefficient sitting in front of the time derivative in (2.2), since the physical meaning of such a heterogeneous coefficient is unclear in Fick's equation (in the heat equation, it would be a heterogeneous product of density by specific heat capacity).

These heterogeneous anisotropic parameters can be approximated by piecewise constant isotropic coefficients, making use of an effective medium approach, as detailed in the supplementary material, which justifies the implementation of multi-layered cylindrical and spherical cloaks with concentric isotropic homogeneous thin layers. The choice of reduced parameters led in Guenneau *et al*. [16] to a multi-layered thermal cloak with piecewise constant and high-contrast diffusivity, and we refer to values of diffusivity and computations therein for the cloaking effect for a concentration of chemical species in two-dimensional setting with 20 layers (there is a one-to-one correspondence between Fourier's heat equation and Fick's equation, the unknown being either the temperature or the concentration). However, figure 1 of the current paper clearly shows cloaking can be achieved with simply five layers with moderate contrast in diffusivity. Moreover, in the spherical case, the choice of reduced parameters leads to a different set of parameters (see the electronic supplementary material) for the multi-layered biocloak, which we now study numerically.

### 2.4. Numerical illustration

**Figure 2.**
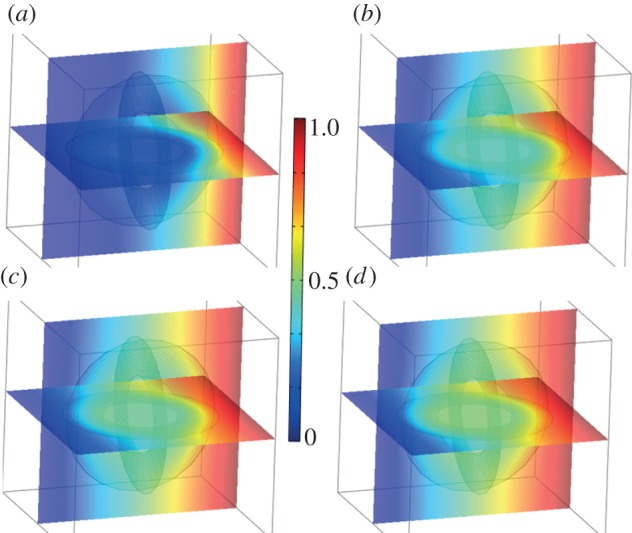
Three-dimensional plot of concentration (mol m^−3^): (*a*) *t* = 0.005 s; (*b*) *t* = 0.01 s; (*c*) *t* = 0.015 s; (*d*) *t* = 0.025 s. It has been checked that three-dimensional plots are as in (*d*) for *t* > 0.025 s (steady state). Spherical cloak of inner radius 1.5 × 10*^−^*^6^ m and outer radius 3.0 × 10*^−^*^6^ m consists of 20 concentric layers with diffusivity ranging from 2.5 × 10*^−^*^6^ to 1.7 × 10*^−^*^2^ m^2^ s*^−^*^1^. The core and outer medium have same diffusivity 1.5 × 10*^−^*^5^ m^2^ s*^−^*^1^. (Online version in colour.)

**Figure 3.**
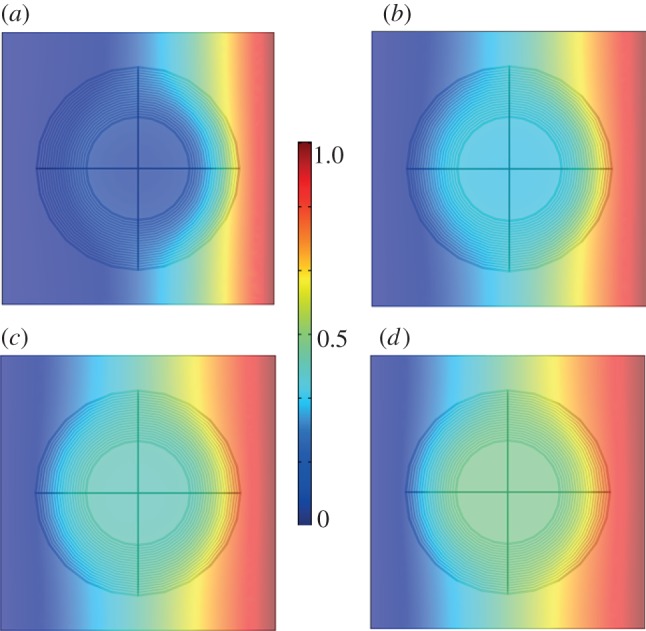
Two-dimensional plot of concentration (mol m^−3^) corresponding to a slice of three-dimensional plot in figure 2 in the horizontal plane passing through the centre of the cloak: (*a*) *t* = 0.005 s; (*b*) *t* = 0.01 s; (*c*) *t* = 0.015 s; (*d*) *t* = 0.025 s. It has been checked that two-dimensional plots are as in (*d*) for time steps *t* > 0.025 s (steady state). (Online version in colour.)

For illustrative purposes, we focus here on a spherical cloak consisting of 20 concentric layers with diffusivity ranging from 2.5 × 10*^−^*^6^ to 1.7 × 10*^−^*^2^ m^2^ s*^−^*^1^, which has been designed for a surrounding medium of diffusivity 1 × 10*^−^*^5^ m^2^ s*^−^*^1^. The inner sphere inside the cloak is also of diffusivity 1 × 10*^−^*^5^ m^2^ s*^−^*^1^. Further details can be found in the supplementary material. In order to emphasize the power of the approach, we consider a cloak substantially larger than that in figure 1. Indeed, as observed in Schittny *et al*. [17] in the context of the transformed heat equation, if we rescale the coordinate system as *x* → *bx* and the time variable *t* → *b*^2^*t* with a dimensionless factor *b*, the transformed Fick's equation (2.2) remains unchanged, provided we assume velocity is ruled out. More precisely, the cloak of figure 1 has been scaled up by a factor *b* = 10^2^, i.e. its inner radius 1.5 × 10*^−^*^6^ m and its outer radius 3.0 × 10*^−^*^6^ m in figure 2. Accordingly, time should be scaled up by a factor *b*^2^ = 10^4^. We show the distribution of concentration for a selection of time points ranging from *t* = 5 × 10*^−^*^3^ s to *t* = 2.5 × 10*^−^*^2^ s in figure 2. Moreover, in figure 2, the cloak is in the presence of a chemical species with concentration normalized to 1 mol m^−3^ for the sake of simplicity (taking any other concentration *C* will simply lead to a colour scale in figures 2 and 3 ranging from 0 to *C* mol m^−3^), which is set on the right-hand side of the computational domain (a cube of sidelength 8.0 × 10*^−^*^6^ m). Note that the cloaking mechanism is preserved if we scale up or down the diffusivities in the inner region, the cloak and the surrounding medium by the same parameter. On the opposite (left-hand) side, we set the usual flux condition (*κ*∇*c*) · ***n*** = *N*_0_ + *k_c_*(*c*_b_−*c*) (with *c* the, as yet unknown, solution to (2.1) and **n** the unit outward normal to each side of the cube), where *κ* varies within the range given in the caption of figure 2 inside the layers of the cloak (for more detail, see the electronic supplementary material), and *κ* = 1 m^2^ s*^−^*^1^ in the inner core and in outside the spherical cloak, and *k_c_* is the mass transfer coefficient, *N*_0_ is the inward flux and *c*_b_ is the bulk concentration of chemical species in the cubic domain. Here, we consider *k_c_* = 5 mol s*^−^*^1^, *N*_0_ = 0 mol m*^−^*^2^ and *c*_b_ = 0 mol m^−3^ s*^−^*^1^. Finally, we set insulation (or equivalent symmetry) conditions 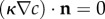 on the four remaining sides of the cube. More detail on implementation of such diffusion models in finite elements may be found in Morton [31], wherein cases of isotropic and anisotropic conductivity are considered (however, this predates transformational optics, so no cloaking is studied there).

This phenomenological model of a biocloak exhibits the following features: the concentration of chemical species nearly vanishes inside the inner sphere of the biocloak at time point *t* = 0.005 s (figure 2*a*). In the optical setting, such a sphere is called invisibility region, as no scattering obstacle placed inside this region can be detected [10–12]. In a biophysical setting, this zone may act as a protection from any potential chemical attack. However, the concentration of chemical species within the inner sphere steadily increases over time until it reaches half the value of the concentration which is set on one side of the cubic computational domain (figure 2*d*). The value of this threshold reached at steady state (from time point *t* = 0.25 s onwards), depends upon the distance from the centre of the cloak to the source: the nearer the cloak from the source, the larger the value of the threshold. We note that the concentration is always uniform inside this inner sphere at any time point (figure 2*a–d*). Such a biocloak might therefore offer some kind of protection from attack of chemical species because the concentration is uniform in its inner sphere at any time point and the concentration therein at any time point is always smaller than it would be without a cloak (see the electronic supplementary material, figure S4). Moreover, it never exceeds a value, which in our configuration is half the applied concentration (it can be seen that this is due to the fact that the cloak has its origin in the centre of the cubic domain).

## 3. Conclusion

In conclusion, in this report, we introduce a coordinate transformation approach to control diffusion processes via anisotropy with an emphasis on concentration of chemical species for potential applications in biophysics or bioengineering. Not only does the form of the transformed convection–diffusion equation involve an anisotropic heterogeneous diffusivity, but it also requires a spatially varying coefficient in front of the time derivative, as well as an anisotropic heterogeneous velocity field. In order to be able to design a structured cloak for such diffusion processes, we have simplified this transformed equation by introducing the so-called reduced coefficients, which preserve the direction of the diffusion flux (but create an impedance mismatch between the cloak and surrounding medium) and by further assuming a small velocity in a homogenization approach. Our theoretical results are exemplified with numerical simulations in two- and three-dimensional settings. It should be emphasized that the issue of convection, which is of particular importance for diffusion in fluids, such as in living organisms, will require further comprehensive studies.
